# Investigating the phenotypic and genetic associations between personality traits and suicidal behavior across major mental health diagnoses

**DOI:** 10.1007/s00406-021-01366-5

**Published:** 2022-02-10

**Authors:** Janos L. Kalman, Tomoya Yoshida, Till F. M. Andlauer, Eva C. Schulte, Kristina Adorjan, Martin Alda, Raffaela Ardau, Jean-Michel Aubry, Katharina Brosch, Monika Budde, Caterina Chillotti, Piotr M. Czerski, Raymond J. DePaulo, Andreas Forstner, Fernando S. Goes, Maria Grigoroiu-Serbanescu, Paul Grof, Dominik Grotegerd, Tim Hahn, Maria Heilbronner, Roland Hasler, Urs Heilbronner, Stefanie Heilmann-Heimbach, Pawel Kapelski, Tadafumi Kato, Mojtaba Oraki Kohshour, Susanne Meinert, Tina Meller, Igor Nenadić, Markus M. Nöthen, Tomas Novak, Nils Opel, Joanna Pawlak, Julia-Katharina Pfarr, James B. Potash, Daniela Reich-Erkelenz, Jonathan Repple, Hélène Richard-Lepouriel, Marcella Rietschel, Kai G. Ringwald, Guy Rouleau, Sabrina Schaupp, Fanny Senner, Giovanni Severino, Alessio Squassina, Frederike Stein, Pavla Stopkova, Fabian Streit, Katharina Thiel, Florian Thomas-Odenthal, Gustavo Turecki, Joanna Twarowska-Hauser, Alexandra Winter, Peter P. Zandi, John R. Kelsoe, Consortium on Lithium Genetics (ConLiGen), PsyCourse, Bernhard T. Baune, Jan Fullerton, Philip B. Mitchell, Peter R. Schofield, Naomi R. Wray, Adam Wright, Susanne A. Bengesser, Eva Reininghaus, Claudio E. M. Banzato, Clarissa Dantas, Martin Alda, Cristiana Cruceanu, Julie Garnham, Paul Grof, Glenda MacQueen, Guy Rouleau, Claire Slaney, Gustavo Turecki, L. Trevor Young, Carlos A. López Jaramillo, Tomás Novák, Pavla Stopkova, Clara Brichant-Petitjean, Mazda Adli, Sébastien Gard, Bruno Etain, Stéphane Jamain, Jean-Pierre Kahn, Marion Leboyer, Mazda Adli, Michael Bauer, Sven Cichon, Franziska Degenhardt, Peter Falkai, Oliver Gruber, Urs Heilbronner, Per Hoffmann, Sarah Kittel-Schneider, Markus Nöthen, Andrea Pfennig, Daniela Reich-Erkelenz, Andreas Reif, Marcella Rietschel, Thomas G. Schulze, Florian Seemüller, Thomas Stamm, Raffaella Ardau, Caterina Chillotti, Maria Del Zompo, Mario Maj, Mirko Manchia, Palmiero Monteleone, Giovanni Severino, Alessio Squassina, Alfonso Tortorella, Kazufumi Akiyama, Kazufumi Akiyama, Ryota Hashimoto, Tadafumi Kato, Ichiro Kusumi, Takuya Masui Takuya Masui, Norio Ozaki, Piotr Czerski, Joanna Hauser, Sebastian Kliwicki, Janusz K. Rybakowski, Maria Grigoroiu-Serbanescu, Alexandru Obregia, Bárbara Arias, Antonio Benabarre, Francesc Colom, Esther Jiménez, Marina Mitjans, Eduard Vieta, Lena Backlund, Lena Backlund, Louise Frisén, Catharina Lavebratt, Lina Martinsson, Urban Ösby, Martin Schalling, Jean-Michel Aubry, Sven Cichon, Alexandre Dayer, Per Hoffmann, Audrey Nallet, Hsi-Chung Chen, David Cousins, Nirmala Akula, Joanna M. Biernacka, Joanna M. Biernacka, Elise T. Bui, J. Ray DePaulo, Sevilla D. Detera-Wadleigh, Mark A. Frye, Fernando S. Goes, Rebecca Hoban, Liping Hou, Layla Kassem, John R. Kelsoe, John R. Kelsoe, Gonzalo Laje, Gonzalo Laje, Susan G. Leckband, Michael J. McCarthy, Francis J. McMahon, Roy H. Perlis, James B. Potash, Thomas G. Schulze, Barbara Schweizer, Lisa R. Seymour, Jordan W. Smoller, Jo Steele, Sarah Tighe, Peter P. Zandi, Eva Reininghaus, Claudio E. M. Banzato, Clarissa Dantas, Martin Alda, Cristiana Cruceanu, Julie Garnham, Paul Grof, Glenda MacQueen, Guy Rouleau, Claire Slaney, Gustavo Turecki, L. Trevor Young, Carlos A. López Jaramillo, Tomás Novák, Pavla Stopkova, Clara Brichant-Petitjean, Bruno Etain, Mazda Adli, Sébastien Gard, Stéphane Jamain, Jean-Pierre Kahn, Marion Leboyer, Mazda Adli, Michael Bauer, Sven Cichon, Franziska Degenhardt, Peter Falkai, Oliver Gruber, Urs Heilbronner, Per Hoffmann, Sarah Kittel-Schneider, Markus Nöthen, Andrea Pfennig, Daniela Reich-Erkelenz, Andreas Reif, Marcella Rietschel, Thomas G. Schulze, Florian Seemüller, Thomas Stamm, Raffaella Ardau, Caterina Chillotti, Maria Del Zompo, Maria Del Zompo, Mario Maj, Mirko Manchia, Palmiero Monteleone, Giovanni Severino, Alessio Squassina, Alfonso Tortorella, Kazufumi Akiyama, Ryota Hashimoto, Ichiro Kusumi, Takuya Masui, Norio Ozaki, Piotr Czerski, Joanna Hauser, Sebastian Kliwicki, Janusz K. Rybakowski, Maria Grigoroiu-Serbanescu, Alexandru Obregia, Bárbara Arias, Antonio Benabarre, Francesc Colom, Esther Jiménez, Marina Mitjans, Eduard Vieta, Lena Backlund, Louise Frisén, Catharina Lavebratt, Lina Martinsson, Urban Ösby, Martin Schalling, Jean-Michel Aubry, Sven Cichon, Alexandre Dayer, Per Hoffmann, Audrey Nallet, Hsi-Chung Chen, David Cousins, Nirmala Akula, Joanna M. Biernacka, Elise T. Bui, J. Ray DePaulo, Sevilla D. Detera-Wadleigh, Mark A. Frye, Fernando S. Goes, Rebecca Hoban, Liping Hou, Layla Kassem, John R. Kelsoe, Gonzalo Laje, Susan G. Leckband, Michael J. McCarthy, Francis J. McMahon, Roy H. Perlis, James B. Potash, Thomas G. Schulze, Barbara Schweizer, Lisa R. Seymour, Jordan W. Smoller, Jo Steele, Sarah Tighe, Peter P. Zandi, Peter Falkai, Udo Dannlowski, Tilo Kircher, Thomas G. Schulze, Sergi Papiol

**Affiliations:** 1 Institute of Psychiatric Phenomics and Genomics (IPPG), University Hospital, LMU Munich, Nussbaumstr. 7, 80336 Munich, Germany; 2 Department of Psychiatry and Psychotherapy, University Hospital Munich, Munich, Germany; 3 International Max Planck Research School for Translational Psychiatry, Munich, Germany; 4 National Center for Global Health and Medicine, Tokyo, Japan; 5 Department of Neurology, Klinikum Rechts Der Isar, School of Medicine, Technical University of Munich, Munich, Germany; 6 Global Computational Biology and Data Sciences, Boehringer Ingelheim Pharma GmbH & Co. KG, 88397 Biberach an der Riß, Germany; 7 Department of Psychiatry, Dalhousie University, Halifax, Canada; 8 Unit of Clinical Pharmacology, University Hospital Agency of Cagliari, Cagliari, Italy; 9 Department of Psychiatry, Geneva University Hospitals, Geneva, Switzerland; 10 Faculty of Medicine, University of Geneva, Geneva, Switzerland; 11 Department of Psychiatry and Psychotherapy, University of Marburg, Marburg, Germany; 12 Center for Mind, Brain and Behavior, University of Marburg, Marburg, Germany; 13 Department of Psychiatric Genetics, Poznan University of Medical Sciences, Poznan, Poland; 14 Department of Psychiatry and Behavioral Sciences, Johns Hopkins University School of Medicine, Baltimore, MD USA; 15 Centre for Human Genetics, University of Marburg, Marburg, Germany; 16 Institute of Human Genetics, School of Medicine &, University of Bonn, University Hospital Bonn, Bonn, Germany; 17 Institute of Neuroscience and Medicine (INM-1), Research Centre Jülich, Jülich, Germany; 18 Psychiatric Genetics Research Unit, Alexandru Obregia Clinical Psychiatric Hospital, Bucharest, Romania; 19 Mood Disorders Clinic of Ottawa, Ottawa, ON Canada; 20 Department of Psychiatry, University of Toronto, Toronto, ON Canada; 21 Institute for Translational Psychiatry, University of Munster, Munster, Germany; 22 Department of Psychiatry and Behavioral Science, Juntendo University Graduate School of Medicine, Tokyo, Japan; 23 Department of Immunology, School of Medicine, Ahvaz Jundishapur University of Medical Sciences, Ahvaz, Iran; 24 Institute for Translational Neuroscience, University of Münster, Munster, Germany; 25 National Institute of Mental Health, Klecany, Czech Republic; 26 3Rd Faculty of Medicine, Charles University, Prague, Czech Republic; 27 Department of Genetic Epidemiology in Psychiatry, Medical Faculty Mannheim, Central Institute of Mental Health, Heidelberg University, Mannheim, Germany; 28 Montreal Neurological Institute, McGill University, Montreal, Canada; 29 Department of Biomedical Sciences, University of Cagliari, Cagliari, Italy; 30 The Douglas Research Centre, McGill University, Montreal, Canada; 31 Department of Psychiatry, University of California San Diego, La Jolla, CA USA; 32 Department of Psychiatry and Behavioral Sciences, SUNY Upstate Medical University, Syracuse, NY USA; 33 Centro de Investigación Biomedica en Red de Salud Mental (CIBERSAM), Barcelona, Spain

**Keywords:** Suicidal behavior, Personality, Polygenic score, Bipolar disorder, Major depression, Schizophrenia

## Abstract

Personality traits influence risk for suicidal behavior. We examined phenotype- and genotype-level associations between the Big Five personality traits and suicidal ideation and attempt in major depressive, bipolar and schizoaffective disorder, and schizophrenia patients (*N *= 3012) using fixed- and random-effects inverse variance-weighted meta-analyses. Suicidal ideations were more likely to be reported by patients with higher neuroticism and lower extraversion phenotypic scores, but showed no significant association with polygenic load for these personality traits. Our findings provide new insights into the association between personality and suicidal behavior across mental illnesses and suggest that the genetic component of personality traits is unlikely to have strong causal effects on suicidal behavior.

**Supplementary Information:**

The online version contains supplementary material available at 10.1007/s00406-021-01366-5.

## Introduction

Suicide is a leading cause of mortality [1], and most individuals with suicidal behavior, which includes suicidal ideation (SI), suicide attempt (SA), and completed suicide, have a diagnosed mental health disorder [2].

Suicidal behavior has a complex, heterogenous etiology. Its risk factors include genetics, personality characteristics, and adverse life events [3]. Twin and family studies showed that suicidal behavior is heritable and that 30–55% of its phenotypic variance is explained by genetic risk factors that only partially overlap with those for mental disease [4–7]. Although individual predictors explain only a fraction of the phenotypic variability, studying them is useful to enhance our understanding of disease pathophysiology and inform the development of diagnostic and preventive measures [8].

Personality characteristics, like the Big Five personality traits (neuroticism, agreeableness, conscientiousness, extraversion, openness), or TEMPS-A temperaments are relatively stable throughout life [9, 10]. They influence the perception of and exposure and response to life events and thus mediate susceptibility and/or resilience to environmental risk factors. For example, individuals with higher neuroticism show high emotional arousal, experience more negative emotions and are more sensitive to negative emotional stimuli and potential loss [10, 11]. In contrast, high extraversion is associated with higher levels of energy and sociability and more positive affect [12]. Hence, multiple studies have provided compelling evidence that high neuroticism and low extraversion are important risk factors for suicidal behavior [13–15]. A substantial amount of the phenotypic variance of these personality traits is explained by common genetic variants: single-nucleotide polymorphism (SNP)-based heritability estimates range from 6 to 15% for neuroticism and 5% to 18% for extraversion [11, 12, 16, 17].

The polygenic makeup of personality traits is one of the few quantifiable biological factors that likely influences suicidal behavior [11, 12, 18], so it is relevant to investigate how much phenotypic variance in suicidal behavior is explained by the polygenic load for personality traits [19]. Furthermore, it would be important to understand whether personality traits have a disease-specific or cross-diagnostic influence on suicidal behavior risk in mental illness. Therefore, we investigated *a)* the association between personality traits and SI and SA across the affective-psychotic diagnosis spectrum, *b)* whether associations differ between diagnostic groups, and *c)* what percentage of phenotypic variation in suicidal behavior is attributable to polygenic scores (PGS) for personality traits.

## Patients and methods

### Sample description

Participants with DSM-IV diagnosis of major depressive disorder (MDD), bipolar disorder (BD), schizoaffective disorder (SCZA), or schizophrenia (SCZ) and available information on lifetime SI or SA (presence/absence) and genetic data were selected from nine independent datasets of European-ancestry cases (*N* = 3012). Sample details, including the definitions of suicidal behavior, are described in the Supplementary Information, including Supplementary Table S4.

The Big Five model is the most widely accepted personality theory, and it is extensively used in research. Two samples (PsyCourse and FOR2107) included individual-level information on the Big Five personality traits, assessed with either the short version of the Big Five Inventory (PsyCourse) or the NEO Five Factor Inventory (FOR2107; Supplementary  Table S6) [20, 21].

### Genetic analyses

The cohorts were genotyped by different microarray types in accordance with local protocols. Quality control and population substructure analyses were performed with PLINK v1.9 and either *R* (for the PsyCourse and FOR2107 cohorts) or the RICOPILI pipeline (for the other seven cohorts), as described previously [22–24] (Supplementary Methods and Supplementary Tables S1-S2)*.* Imputation was performed with SHAPEIT and IMPUTE2 with the 1000 Genomes Phase 3 (for the FOR2107 and PsyCourse cohorts) or the Haplotype Reference Consortium v1.0 (for the other cohorts) reference panels. For our analyses, we selected variants present in the PRS-CS 1000 Genomes Phase 3 EUR reference dataset.

PRS-CS was used to calculate PGS for personality traits with significant effects at the phenotype level by using summary statistics from genome-wide association studies as training datasets (Supplementary Table S3) [11, 12, 25]*.*

### Statistical analyses

In the primary phenotype-level analyses, we analyzed the association of personality traits with SI and SA within the PsyCourse and FOR2017 samples by logistic regression, with sex, age, and BD subtype as covariates. Results of these analyses were meta-analyzed using fixed- and random-effects inverse variance-weighted meta-analyses. Potential subgroup effects specific for DSM categories were investigated with diagnosis-specific meta-analyses. To investigate associations of extraversion and neuroticism PGS with SI and SA, we used the same analysis models as for the phenotype-level analyses. Genotyping batch (for the Romania1 and euoR samples) and the first eight multidimensional scaling ancestry components were used as additional covariates. The statistical power of our sample was estimated with G*Power 3.1 and the *avengeme* R package [26, 27]. The significance threshold was corrected for 14 tests by Bonferroni’s method (α = 0.05/[5 personality traits × 2 phenotypes and 2 PGS × 2 phenotypes] = 3.57 × 10^–3^).

## Results

The frequency of SI and SA in our study was 61.16% and 31.28%, respectively.

### Personality and suicidal behavior

Our study had 80% power (α = 0.05) to detect effects of personality traits with odds ratio (OR) ≥ 1.15 on SI and OR ≥ 1.23 on SA.

**Fig. 1:**
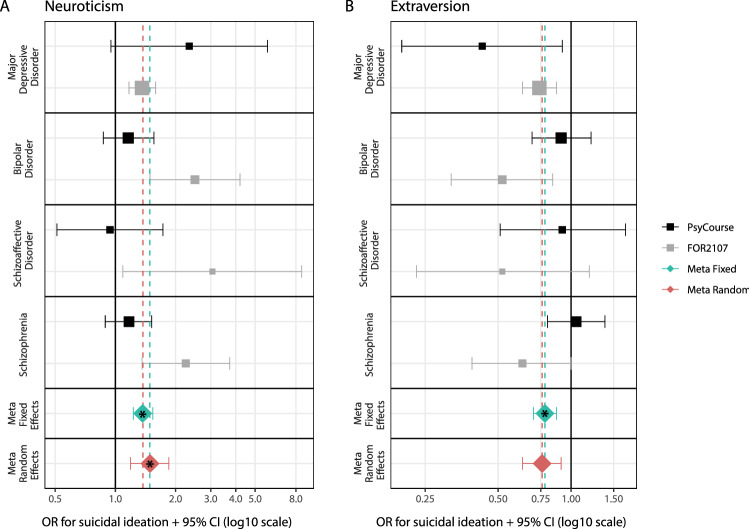
Results of secondary phenotype-level analyses. For extraversion and neuroticism, which were significantly associated with suicidal ideation in the primary analyses, we conducted secondary analyses to investigate potential differences across the diagnostic spectrum. Results of the individual regression models can be found in Supplementary Table S7. Inverse variance-weighted meta-analysis *p* values that were significant after Bonferroni correction (α < 3.57 × 10^–3^) are indicated with an asterisk. Meta fixed, inverse variance-weighted fixed-effects meta-analysis; meta random, inverse variance-weighted random-effects meta-analysis; 95% CI, 95% confidence intervals (the 95% CIs were constrained to a minimum of 0 and a maximum of 1); OR, odds ratio (a higher OR indicates an association with suicidal ideation

In the fixed-effects meta-analysis of the FOR2107 and PsyCourse samples, neuroticism was significantly associated with an increased likelihood of SI (OR = 1.37, 95% CI [1.23–1.54], *p* = 2.11 × 10^−8^, Cochran’s *Q* *p* = 0.02, *I*^2^ = 57.9%), and extraversion, with a decreased likelihood (OR = 0.78, 95% CI [0.70–0.87], *p* = 1.01 × 10^−5^, Cochran’s *Q* *p* = 0.06, *I*^2^ = 48.3%) *(*Table 1, Fig. 1). The random-effects meta-analysis results were not substantially different (Table 1). In the secondary, diagnosis-specific meta-analyses, the effect direction was consistent across diagnostic groups, although a high level of heterogeneity (Cochran’s *Q* *p* < 0.05) was observed in all diagnoses except MDD and partially SCZA (Supplementary Table S7). After correction for multiple testing, none of the other personality traits showed significant associations (Table 1).

**Table 1:** Results of the (A) primary phenotype-level and (B) polygenic score analyses

(A) Analyses of the associations of the Big Five personality traits with suicidal ideation and suicide attempt											
Personality trait	Meta	Suicidal ideation (SI)					Suicide attempt (SA)				
		*N* (SI + /SI−)	OR	95% CI	*p*	Cochran’s *Q* *p*	*N* (SA + /SA−)	OR	95% CI	*p*	Cochran’s *Q* *p*
Agreeableness	FE	1786 (1294/492)	0.86	0.77–0.96	6.99 × 10^−3^	0.20	961 (324/637)	0.98	0.85–1.13	0.74	0.15
	RE		0.84	0.72–0.98	0.02			0.94	0.76–1.16	0.55	
Conscientiousness	FE		0.92	0.82–1.02	0.11	0.94		1.02	0.88–1.18	0.80	0.06
	RE		0.92	0.82–1.02	0.11			0.99	0.78–1.27	0.95	
Extraversion	FE		0.78	0.70–0.87	**1.01 × 10**^**−5**^	0.06		0.93	0.81–1.07	0.31	0.08
	RE		0.76	0.63–0.91	3.77 × 10^−3^			0.86	0.68–1.09	0.21	
Neuroticism	FE		1.37	1.23–1.54	**2.11 × 10**^**−8**^	0.02		1.14	0.99–1.32	0.07	0.06
	RE		1.49	1.19–1.85	**3.82 × 10**^**−4**^			1.22	0.96–1.55	0.11	
Openness	FE		1.01	0.90–1.12	0.91	0.49		0.99	0.86–1.14	0.86	0.33
	RE		1.01	0.90–1.12	0.91			1	0.85–1.17	0.99	

(B) Analyses of the associations of polygenic scores for extraversion and neuroticism with suicidal ideation and suicide attempt											
Personality trait	Meta	Suicidal ideation (SI)					Suicide attempt (SA)				
		*N* (SI+/SI−)	OR	95% CI	*p*	Cochran’s *Q* *p*	N (SA+/SA−)	OR	95% CI	*p*	Cochran’s *Q* *p*
Extraversion	FE	3012 (1842/1170)	1.07	0.98–1.17	0.12	0.70	2180 (682/1498)	1.04	0.94–1.15	0.44	0.12
	RE		1.07	0.98–1.17	0.12			1.04	0.91–1.19	0.55	
Neuroticism	FE		0.90	0.83–0.99	0.02	0.07		0.95	0.86–1.05	0.30	0.02
	RE		0.89	0.78–1	0.07			0.92	0.79–1.07	0.30	

In the primary phenotype-level analyses, the association of personality traits with suicidal ideation and suicide attempt was analyzed by logistic regression in the PsyCourse and FOR2017 samples. Results of these analyses were meta-analyzed by both fixed- and random-effects inverse variance-weighted meta-analyses. To investigate the associations of polygenic scores for extraversion and neuroticism (personality traits that showed significant effects at the phenotype level) with SI and SA, we used the same analysis models as for the phenotype-level analyses.

*Meta,* inverse variance-weighted meta-analysis, *FE* fixed effects, *RE* random effects, *N* total sample size, *SI+/SI− and SA+/SA−* the number of patients with and without suicidal ideation (SI + and SI−, respectively) and with or without suicide attempt (SA +  and SA−, respectively), *OR* odds ratio (a higher OR indicates an association with suicidal ideation or suicide attempt, *95% CI* 95% confidence interval (the 95% CIs were constrained to a minimum of 0 and a maximum of 1), *p* unadjusted *p* value (significance threshold corrected for multiple testing by Bonferroni’s method: α = 3.57 × 10^–3^; *p* values that were significant after Bonferroni correction are indicated in bold font)

Note: Polygenic scores were calculated with summary statistics from the latest genome-wide association studies of the respective traits as training datasets [11, 12]

None of the personality traits was significantly associated with SA, although the direction of the effects was the same as with SI (Table 1).

### PGS for personality traits and suicidal behavior

No significant association was found between neuroticism and extraversion PGS and SI or SA (Table 1). Post hoc power analyses indicated that none of the PGS analyses in our sample had 80% power to identify PGS effects with *p* < 0.05.

## Discussion

To our knowledge, this study is the first attempt to dissect the phenotypic and genetic relationship between personality traits and suicidal behavior across the affective-psychotic diagnostic spectrum. We found significant phenotype-level associations of both neuroticism and extraversion—two personality traits known to influence affect processing—with SI across diagnostic groups but no evidence that these associations were driven by the polygenic load for these traits.

An association of neuroticism with increased suicidal behavior risk has already been described in population-based cohorts [14, 28, 29] and studies on individuals with personality [30] or affective disorders [31–33]; our secondary analyses confirmed these findings in patients with MDD (the largest diagnostic group in our study) and showed similar effects for BD and SCZ, suggesting that neuroticism may represent a transdiagnostic risk factor for SI [34].

Studies reported a protective effect of extraversion in the general population [15, 35] and patients with affective disorder [33, 35]. The diagnosis-specific results in our study support such a protective effect in MDD and suggest similar effects in BD.

Although our SA sample was sufficiently powered to detect effect sizes comparable to those observed for SI, we found no significant associations of neuroticism and extraversion with SA, which is a more severe phenotype than SI. This finding suggests a lesser involvement, or a lack thereof, of personality traits in SA in comparison with SI.

The present study constitutes, to our knowledge, the first attempt to ascertain the role of a polygenic load associated with personality traits on suicidal behavior in a sample exclusively composed of patients with psychiatric diagnoses. Despite the phenotype-level associations, neuroticism and extraversion PGS were not significantly associated with suicide-related phenotypes, which contrasts with a study that detected an association between neuroticism PGS and SA or self-harm in a population-based cohort of 4959 individuals [36]. However, according to our post-hoc analysis, our study lacked statistical power to replicate these findings. Furthermore, neuroticism and extraversion PGS explained only a small proportion of the phenotypic variance of the respective personality traits in our study (*R*^2^_neuroticism_ = 0.011, *R*^2^_extraversion_ = 0.0059).

## Limitations

The heterogeneous definitions of personality, SI, and SA in the samples, as also implied by the heterogeneity estimates (*I*^2^) of our analyses, are an important limitation. SI and SA are broad concepts with no universally accepted definitions [37]. Accordingly, their prevalence might be impacted by cohort-specific differences, as also observed in our study (Supplementary Tables S4, S6). A further potential source of heterogeneity was the use of different questionnaires to assess personality traits in the various cohorts. Notably, these issues represent a general problem in psychiatric research [38]. To assess the effect of heterogeneity on our results, we performed random- and fixed-effects meta-analyses. Another limitation is that we did not account for possible confounders by assessing environmental precipitating factors. Last, our sample had limited statistical power, which reduced the likelihood of detecting true-positive signals.

## Conclusion

Our findings reinforce the notion that personality traits contribute to the expression of SI independently of diagnosis, and they provide preliminary evidence that personality trait PGS are unlikely to have strong causal effects on suicidal behavior. These findings need validation in larger clinical datasets.

## Supplementary Information

Below is the link to the electronic supplementary material.

Supplementary file1 (PDF 671 KB)
