# Supplementary material for: Investigating the phenotypic and genetic associations between personality traits and suicidal behavior across major mental health diagnoses
Source: Eur Arch Psychiatry Clin Neurosci. 2022 Feb 10;272(8):1611–20. doi: 10.1007/s00406-021-01366-5 (PMC9653330; doi:10.1007/s00406-021-01366-5)
Supplement: Supplementary file 1 — Supplementary file1 (PDF 671 KB) [file 406_2021_1366_MOESM1_ESM.pdf]

European Archives of Psychiatry and Clinical Neuroscience

Investigating the phenotypic and genetic association between personality and suicidal behavior across major mental health diagnoses

Online Resource

Janos L. Kalman[1,2,3], Tomoya Yoshida[4], Till F. M. Andlauer[5,6], Eva C. Schulte[1,2], Kristina Adorjan[1,2], Martin Alda[7], Raffaela Ardau[8]; Jean-Michel Aubry[9,10]; Katharina Brosch[11,12], Monika Budde[1]; Caterina Chillotti[8], Piotr M. Czerski[13]; Raymond J. DePaulo[14]; Andreas Forstner[15,16,17], Fernando S. Goes[14]; Maria Grigoroiu-Serbanescu[18]; Paul Grof[19,20]; Dominik Grotegerd[21], Tim Hahn[21], Maria Heilbronner[1], Roland Hasler[9]; Urs Heilbronner[1], Stefanie Heilmann-Heimbach[16], Pawel Kapelski[13]; Tadafumi Kato[22]; Mojtaba Oraki Kohshour[1,23]; Susanne Meinert[21,24], Tina Meller[11,12], Igor Nenadić[11,12], Markus M Nöthen[16], Tomas Novak[25,26]; Nils Opel[21]; Joanna Pawlak[13]; Julia-Katharina Pfarr[11,12]; James B. Potash[14], Daniela Reich-Erkelenz[1], Jonathan Repple[21], Hélène Richard-Lepouriel[9]; Marcella Rietschel[27], Kai G. Ringwald[11,12], Guy Rouleau[28]; Sabrina Schaupp[1], Fanny Senner[1,2], Giovanni Severino[29], Alessio Squassina[29], Frederike Stein[11,12], Pavla Stopkova[25,26]; Fabian Streit[27], Katharina Thiel[21] ; Florian Thomas-Odenthal[11]; Gustavo Turecki[30]; Joanna Twarowska-Hauser[13]; Alexandra Winter[21]; Peter P. Zandi[14]; Consortium on Lithium Genetics (ConLiGen), FOR2107, PsyCourse, Peter Falkai[2]; Udo Dannlowski[21], Tilo Kircher[11,12], Thomas G. Schulze[1,14,27,31,32], Sergi Papiol[1,2,33]

[+] Thomas G. Schulze and Sergi Papiol are joint senior authors

Corresponding Author:

Janos L. Kalman, MD
Institute of Psychiatric Phenomics and Genomics (IPPG),
University Hospital, LMU Munich,
Nussbaumstr. 7
80336 Munich, Germany
Tel: +49-89-4400-53403
Fax: +49-89-4400-55547
Email: janos.kalman@med.uni-muenchen.de

Table of Contents

Description of the individual study samples ... 3

1. EuropeR | Czech Republic, Switzerland ... 3
2. FOR2107 ... 3
3. Halifax ... 3
4. Italy | Italy ... 3
5. Poland | Poland ... 4
6. PsyCourse ... 4
7. Romania1 ... 4
8. Romania2 ... 5
9. San Diego | USA ... 5

Supplementary Methods ... 6

Supplementary Note 1. Calculation of multidimensional scaling ancestry components ... 6

Supplementary Tables ... 7

Supplementary Table S1. References for published methods ... 7

Supplementary Table S2. Genetic quality control filters ... 8

Supplementary Table S3. Parameters used to calculate polygenic scores ... 9

Supplementary Table S4. Overview of the scales used to measure suicidal ideation and suicide attempt by the individual cohorts ... 9

Supplementary Table S5. Sample characteristics ... 10

Supplementary Table S6. Distribution of the Big Five personality scale scores ... 11

Supplementary Table S7. Results of the secondary phenotype-level analyses ... 12

Funding, conflict of interest and acknowledgements ... 13

References for the Online Resource ... 16

Description of the individual study samples

The list below provides a detailed description (based on the original publications [1–3]) of all cohorts that were part of the present study.

1. EuropeR | Czech Republic, Switzerland

For the current study, datasets from the following study sites were combined and analyzed together:
Czech subsample (n = 44): Unrelated patients were recruited at in- and outpatient units at the Prague Psychiatric Center, Psychiatric Hospital Bohnice, Psychiatric Clinic, Czech Republic. Diagnoses were made on the basis of either a Schedule for Affective Disorders and Schizophrenia-Lifetime (SADS-L) interview or an unstructured clinical interview modified from SADS-L by using Research Diagnostic Criteria (RDC) criteria. All patients signed an informed consent form approved by the institutional review boards (IRBs) of the Prague Psychiatric Center.
Swiss subsample (n = 43): Patients with bipolar disorder (BD) were recruited at the specialized outpatient unit for mood disorders at the Division of Psychiatric Specialties of the Department of Psychiatry in Geneva, Switzerland. Patients are referred to this unit by psychiatrists or general practitioners for diagnostic assessment and care. Individuals with a Diagnostic and Statistical Manual of Mental Disorders, fourth edition (DSM-IV) diagnosis of BD type I (BD-I) or type II (BD-II) diagnosed by a trained psychiatrist or clinical psychologist were included in this study. Clinical and anamnestic data (medical histories, family history, onset of the disorder, and previous treatments) were collected during the interview. Patients with BD were evaluated with the French version of the Diagnostic Interview for Genetic Studies (DIGS) or the French version of the Structured Clinical Interview for DSM-IV Axis I Disorders (SCID I, version 2.0). They were also evaluated with the DIGS for comorbid Axis I disorders. Treatment response to lithium was evaluated with the Alda scale. All patients completed self-report questionnaires, including the Barratt Impulsiveness Scale (BIS-10), State Anger Expression Inventory (STAXI), Beck Hopelessness Scale (BHS), Childhood Trauma Questionnaire (CTQ), Brown-Goodwin Aggression Scale (BGA), and the Geneva Suicide History Form. All patients provided written informed consent.

2. FOR2107

In- and outpatients (aged 18-65 years) with a diagnosis of BD, major depressive disorder (MDD), schizophrenia (SZ), or schizoaffective disorder (SCZA), as well as healthy individuals, were recruited between 2014 and 2018 as part of a multi-center (Universities of Marburg and Münster, Germany) longitudinal cohort study (DFG research group FOR2107, www.for2107.de) [2] and followed up for two years. Trained psychologists conducted semi-structured SCID-I interviews and collected behavioral, psychophysiological, neural, and (epi)genetic data. The study protocols were approved by the ethics committees of the Medical Schools of the Universities of Marburg and Münster, and the studies were performed in accordance with the Declaration of Helsinki, and all participants provided written informed consent.

3. Halifax

The case samples were recruited from patients longitudinally followed at specialty mood disorders clinics in Halifax and Ottawa (Canada). Patients were interviewed in a blind fashion with the SADS-L, and consensus diagnoses were made according to the DSM-IV and RDC. Protocols and procedures were approved by the local ethics committees, and written informed consent was obtained from all patients before participation in the study.

4. Italy | Italy

The sample comprised unrelated patients with BD. All patients were of Sardinian ancestry for at least 3 generations. They were recruited at the outpatient unit (Lithium Clinic) of the Clinical Psychopharmacology Center at the Department of Biomedical Science, Section of Neuroscience &

Clinical Pharmacology, University of Cagliari, Cagliari, Italy, and Unit of Clinical Pharmacology, University Hospital Agency of Cagliari, Cagliari, Italy. Lifetime consensus diagnoses according to RDC were made by trained clinical psychopharmacologists on the basis of data from a personal semi-structured interview and a systematic review of patients' medical records. Informed written consent to participate in the study was obtained from all patients. The study was approved by the local ethics committee.

5. Poland | Poland

Patients were recruited at the Department of Psychiatry, Poznan University of Medical Sciences, Poznan, Poland. All patients received a lifetime diagnosis of BD according to DSM-IV criteria on the basis of a consensus best-estimate procedure and structured diagnostic interviews with the Structured Clinical Interview for DSM-IV Disorders (SCID). Study protocols were reviewed and approved in advance by the IRBs of the participating institutions. All patients provided written informed consent.

6. PsyCourse

The samples were part of a multi-site German/Austrian longitudinal study (www.psycourse.de) that was conducted between 1 January 2012 and 31 December 2019 [3] and collected deep phenotypic, neuropsychologic, and omics data from patients with brief psychotic disorder, MDD, BD, SCZ, and SCZA and healthy individuals. Adult participants were referred from the clinical staff or identified by querying patient registries. Diagnoses were made according to DSM-IV. Study protocols were reviewed and approved by the ethics committees of the University Medical Center Göttingen (UMG Göttingen, Bad Zwischenahn, Eschwege, Asklepios Specialized Hospital Göttingen, Hildesheim, Lüneburg, Liebenburg, Osnabrück, Rotenburg, Tiefenbrunn, Wilhemshaven), Medical Faculty of the LMU Munich (Munich and Augsburg), Medical Faculty of the RU Bochum (Bochum), Medical Association Bremen (Bremen Ost), Medical University of Graz (Graz), Ulm University (Günzburg), Medical Association Westfalen-Lippe, and Medical Faculty University of Münster (Münster), and the studies were performed in accordance with the Declaration of Helsinki. All participants provided written informed consent. The current analyses were based on the v4.1 version of the data set.

7. Romania1

rom3: Patients with BD-I were recruited between 2003 and 2018 from consecutive admissions to the Obregia Clinical Psychiatric Hospital, Bucharest, Romania. Patients were interviewed with the DIGS and Family Interview for Genetic Studies (FIGS). Information was also obtained from medical records and close relatives. The diagnosis of BD-I was assigned according to DSM-IV criteria on the basis of the best estimate procedure. All patients had at least two hospitalizations for illness episodes. All participants provided written informed consent. The study was performed in accordance with the ethical principles of the Declaration of Helsinki.

bmrom: This sample includes the BOMA-Romania sample and additional cases from the ConLiGen-Romania sample. Patients with BD-I were recruited between 2003 and 2018 from consecutive admissions to the Obregia Psychiatric Hospital of Bucharest, Romania. Patients were interviewed with the DIGS and FIGS. Information was also obtained from medical records and close relatives. The diagnosis of BD-I was assigned according to DSM-IV criteria on the basis of both the DIGS and medical records. Patients were included in the sample if they had at least two documented hospitalizations for illness episodes (one manic/mixed and one depressive or two manic episodes). This information was also confirmed by first degree relatives for 64% of the patients. All participants provided written informed consent. The study was performed in accordance with the ethical principles of Declaration of Helsinki. Patients in the ConLiGen-Romania study were recruited in the same manner as the patients in the BOMA-Romania sample and were required to have taken lithium for at least 2 years; lithium treatment response was evaluated with the Alda scale.

Because of the similarities in sample ascertainment and the genotyping microarrays used (Illumina OmniExpress in both samples), patients in the *rom3* and *bmrom* cohorts were merged. Quality control, population substructure analyses, and imputation were performed on the combined dataset.

8. Romania2

Patients were recruited at the Obregia Clinical Psychiatric Hospital, Bucharest, Romania, as described for the *bmrom* sample above.

9. San Diego | USA

Genetic Association Information Network (GAIN)/ The Bipolar Genome Study (BiGS) The BD sample was collected under the auspices of the NIMH Genetics Initiative for BD (http://zork.wustl.edu/nimh/), genotyped as part of GAIN and analyzed as part of a larger genome-wide association study conducted by the BiGS consortium. Approximately half of the GAIN sample was collected as multiplex families or sib-pair families (waves 1-4), and the remainder was collected as individual cases (wave 5). Patients were recruited at 11 sites: Indiana University; Johns Hopkins University; the NIMH Intramural Research Program; Washington University at St. Louis; University of Pennsylvania; University of Chicago; Rush Medical School; University of Iowa; University of California, San Diego; University of California, San Francisco; and University of Michigan. All investigations were carried out after the protocols had been reviewed by the IRB at each participating institution. At all sites, potential patients were identified by screening admissions to local treatment facilities and through publicity programs or advocacy groups and evaluated with the DIGS, FIGS, and information from relatives and medical records. All information was reviewed through a best-estimate diagnostic procedure by 2 independent and non-interviewing clinicians, who reached a best-estimate diagnosis. In the event of a disagreement, a third independent, non-interviewing clinician performed a review to break the tie.

Supplementary Methods

Supplementary Note 1. Calculation of multidimensional scaling ancestry components

For the population substructure analysis, pre-imputation genotype data were used after quality control (Supplementary Table S1).
Additional variant filtering steps for this analysis were as follows:
Removal of variants with a minor allele frequency < 0.05 or Hardy-Weinberg equilibrium test *p* value $<10^{-3}$; removal of variants mapping to the extended major histocompatibility complex region (chromosome 6, 25-35 Mbp) or to a typical inversion site on chromosome 8 (7-13 Mbp); linkage disequilibrium pruning (command *--indep-pairwise 200 100 0.2*).
Next, the pairwise identity-by-state (IBS) matrix of all individuals was calculated by using the command *--genome* on the filtered genotype data. Multidimensional scaling (MDS) analysis was performed on the IBS matrix with the eigendecomposition-based algorithm in PLINK v1.9.
MDS ancestry components calculated separately for each cohort were used as covariates in genetic analyses.

Supplementary Tables

Supplementary Table S1. References for published methods

Method / Tool	Reference
PGC Rapid Imputation and Computational Pipeline for GWAS (RICOPILI)	Lam et al. 2020 [4]
SHAPEIT	Delaneau et al. 2013 [5]
IMPUTE2	Howie et al. 2009, Howie et. al 2012 [6, 7]
PLINK	Chang et al. 2015 [8]
Haplotype Reference Consortium v1.0 reference panel	McCarthy et al. 2016 [9]
PRS-CS	Ge et al. 2019 [10]

Supplementary Table S2. Genetic quality control filters

Overview of the genetic quality control exclusion criteria for the removal of variants and individuals.

		FOR2107	PsyCourse	ConLiGen
**Genotyping array**		PsychChip	PsychChip	see *Note*
**QC**	SNV call rate (%)	< 98	< 98	< 98
	SNV MAF (%)	< 1	< 1	< 1
	Genotyping rate (%)	< 98	< 98	< 98
	Removal of sex mismatches	Yes	Yes	Yes
	Removal of non-autosomal variants	Yes	Yes	Yes
	Removal of cryptic relatives (pi-hat, %)	≥12.5	≥12.5	> 20
	Genetic outliers (MDS)	> 4 SD	> 4 SD	> 4 SD
	Hardy-Weinberg equilibrium test *p* value	$< 1\times10^{-6}$	$< 1\times10^{-6}$	$< 1\times10^{-10}$
**Imputation reference panel**		1000 Genomes phase 3	1000 Genomes phase 3	HRC v1.0
**QC**	SNV MAF (%)	< 1	< 1	< 1
	IMPUTE2 INFO metric	< 0.80	< 0.80	< 0.80

Abbreviations: HRC, Haplotype Reference Consortium; MAF, minor allele frequency; MDS, multidimensional scaling analysis of the pairwise genetic relationship matrix; PsychChip, Illumina PsychArray BeadChip; QC, quality control; SNV, single nucleotide variant

Note: From pairs of genetic relatives across all four samples, only the individual with the most complete phenotypic information on suicidal behavior, age, sex, and diagnosis was retained. The ConLiGen samples were genotyped on the following microarrays: Illumina OmniExpress (EuropeR, Italy, Halifax, Romania1, San Diego), I610Q and I660Q (Poland), and Illumina Global Screening Array (Romania2).

Supplementary Table S3. Parameters used to calculate polygenic scores

Overview of the genome-wide association studies (GWASs) used as training data in the calculation of polygenic scores and the PRS-CS global shrinkage parameter (φ) values (determined automatically) across the GWASs and cohorts.

	**Extraversion** [11]		**Neuroticism** [12]	
	GWAS sample size	φ	GWAS sample size	φ
**EuropeR**	63,030	1.03×10^{-4}	329,821	1.40×10^{-4}
**FOR2107**		1.04×10^{-4}		1.36×10^{-4}
**Halifax**		1.03×10^{-4}		1.40×10^{-4}
**Italy**		1.03×10^{-4}		1.40×10^{-4}
**Poland**		1.03×10^{-4}		1.40×10^{-4}
**PsyCourse**		9.96×10^{-5}		1.40×10^{-4}
**Romania1**		1.01×10^{-4}		1.38×10^{-4}
**Romania2**		1.01×10^{-4}		1.38×10^{-4}
**SanDiego**		1.03×10^{-4}		1.40×10^{-4}

Abbreviations: φ, global shrinkage parameter (determined automatically); GWAS, genome-wide association study

Note: The PGC Romania1 GWAS sample consisted of 2 cohorts, rom3 and bmrom, which were merged and analyzed together in the present study because of the similarities in sample ascertainment and the genotyping microarrays used (both Illumina OmniExpress).

Supplementary Table S4. Overview of the scales used to measure suicidal ideation and suicide attempt by the individual cohorts

**Cohort**	**Measurement**
EuropeR	*Czech subsample:* SADS-L<br>*Swiss subsample:* DIGS, Geneva Suicide History Form
FOR2107	OPCRIT (suicidal ideation), Suicide Behaviors Questionnaire-Revised (suicide attempt)
Halifax	SADS-L
Italy	SADS-L
Poland	Questionnaire on suicidal behavior as an appendix to SCID-I
PsyCourse	SCID
Romania1	DIGS, FIGS, review of medical records, and information from relatives
Romania2	DIGS, FIGS, review of medical records, and information from relatives
SanDiego	SADS for some subjects, DIGS for others

Abbreviations: SADS-L, Schedule for Affective Disorders and Schizophrenia lifetime version; DIGS, Diagnostic Interview for Genetics Studies; OPCRIT, Operational Criteria Checklist for Psychotic Illness and Affective Illness; SCID, Structured Clinical Interview for DSM-IV-TR; FIGS, Family Interview for Genetics Studies

Supplementary Table S5. Sample characteristics

						Suicidal ideation *		Suicide attempt **	
**Diagnosis**	**Cohort**	**Age,** mean (SD)	**Sex,** % male	**Duration of illness,** median (MAD)	**Diagnosis,** % BD-I	**N**	**% yes**	**N**	**% yes**
**MDD**	FOR2107	36.60 (13.22)	35.50	8 (7.41)		814	68.92	204	25.49
	PsyCourse	41.33 (15.14)	43.06	8.5 (8.15)		72	81.94	72	20.83
	**Meta**	**36.98 (13.44)**	**36.12**	**8 (7.41)**		**886**	**69.98**	**276**	**24.28**
**BD**	EuropeR	45.37 (12.20)	40.23	17 (13.34)	81.61	87	74.71	87	49.43
	FOR2107	42.60 (13.04)	48.82	14 (11.86)	59.06	127	76.38	27	51.85
	Halifax	48.03 (12.75)	42.31	19 (13.34)	69.23	130	28.46	130	26.92
	Italy	46.17 (14.83)	31.22	17 (10.38)	74.07	189	35.45	189	21.69
	Poland	62.34 (10.02)	37.50	30.5 (12.60)	87.50	80	23.75	80	23.75
	PsyCourse	45.60 (12.97)	50.40	13 (11.86)	77.90	371	80.86	361	34.35
	Romania1	41.71 (12.95)	42.27	13 (10.37)	100	388	35.57	388	26.55
	Romania2	42.28 (12.73)	28.12	13.5 (9.64)	100	96	26.04	96	30.21
	SanDiego	45.35 (13.61)	48.45	25 (14.83)	89.69	97	81.44	92	51.09
	**Meta**	**45.29 (13.76)**	**42.56**	**16 (11.86)**	**83.45**	**1565**	**52.84**	**1450**	**31.38**
**SCZA**	FOR2107	39.23 (12.54)	49.06	16 (8.89)		53	86.79	15	33.33
	PsyCourse	45.83 (10.42)	37.04	16 (11.86)		81	77.78	81	35.80
	**Meta**	**43.21 (11.72)**	**41.79**	**16 (11.12)**		**134**	**81.34**	**96**	**35.42**
**SCZ**	FOR2107	38.52 (11.06)	62.37	15 (11.86)		93	53.76	27	29.63
	PsyCourse	40.38 (11.95)	64.97	13 (11.86)		334	70.66	331	35.65
	**Meta**	**39.97 (11.78)**	**64.4**	**13 (10.38)**		**427**	**66.98**	**358**	**35.2**

Abbreviations: BD, bipolar disorder; BD-I, bipolar disorder type I; MAD, median absolute deviation; MDD, major depressive disorder; SD, standard deviation; SCZ, schizophrenia; SCZA, schizoaffective disorder.

Note: See the "Description of the individual study samples" section of the Supplementary Material for detailed sample descriptions. The median absolute deviation was calculated by using 1.4826 as a constant.

* The age, sex, duration of illness, and diagnosis columns of the table show the characteristics of patients with available data on suicidal ideation.

** Information on suicide attempt was not available for all patients.

Supplementary Table S6. Distribution of the Big Five personality scale scores

		N	**Agreeableness,** median (MAD)	**Conscientiousness,** median (MAD)	**Extraversion,** median (MAD)	**Neuroticism,** median (MAD)	**Openness,** median (MAD)
**MDD**	FOR2107	792	0.09 (0.98)	-0.06 (1)	0.037 (0.97)	0.11 (1.03)	0.06 (1.03)
	PsyCourse	62	-0.32 (0.86)	0.29 (0.82)	-0.16 (0.78)	0.41 (1.14)	0.07 (0.80)
**BD**	FOR2107	123	0.15 (0.92)	0.06 (0.97)	0.14 (0.91)	0.09 (1.15)	0.08 (1.07)
	PsyCourse	329	0.09 (0.9)	-0.12 (0.85)	0.25 (1.37)	-0.13 (1.45)	0.29 (1.47)
**SCZA**	FOR2107	48	-0.01 (0.96)	-0.02 (0.77)	0.01 (0.97)	0.02 (1.21)	0.1 (1.05)
	PsyCourse	67	0.11 (0.91)	-0.13 (0.87)	0.16 (1.41)	0.19 (0.85)	0.02 (0.79)
**SCZ**	FOR2107	92	-0.1 (0.9)	-0.03 (0.9)	-0.12 (0.92)	0.12 (0.98)	-0.03 (1)
	PsyCourse	365	-0.09 (0.95)	-0.06 (0.9)	-0.24 (0.76)	-0.22 (0.79)	-0.03 (1.46)

Abbreviations: BD, bipolar disorder; MAD, median absolute deviation; MDD, major depressive disorder; SD, standard deviation; SCZ, schizophrenia; SCZA, schizoaffective disorder.

Notes: Personality traits were available only for a subset of the study participants and were assessed with either the short version of the Big Five Inventory (PsyCourse) or the NEO Five Factor Inventory (FOR2107). To facilitate comparisons, the scores were scaled within each cohort. The median absolute deviation was calculated by using 1.4826 as a constant.

Supplementary Table S7. Results of the secondary phenotype-level analyses

For extraversion and neuroticism, which were significantly associated with suicidal ideation in the primary analyses, we conducted secondary analyses to investigate potential differences across the diagnostic spectrum.

			**Extraversion**					**Neuroticism**				
**Diagnosis**	**Cohort**	**N**	**OR**	**95% CI**	***p***	**I^2 %**	**Cochran's Q p**	**OR**	**95% CI**	***p***	**I^2 %**	**Cochran's Q p**
**MDD**	FOR2107	792	0.74	0.63-0.87	1.6×10^{-4}			1.36	1.17-1.59	6.81×10^{-5}		
	PsyCourse	62	0.43	0.20-0.93	0.03			2.34	0.95-5.77	0.06		
	**FE Meta**	854	0.72	0.62-0.84	3.42×10^{-5}	46.3	0.17	1.39	1.19-1.61	2.28×10^{-5}	25	0.24
	**RE Meta**		0.65	0.41-1.02	0.06			1.48	1.02-2.15	0.04		
**BD**	FOR2107	123	0.52	0.32-0.84	7.2×10^{-3}			2.50	1.49-4.20	5.47×10^{-4}		
	PsyCourse	329	0.91	0.69-1.21	0.53			1.16	0.87-1.56	0.3		
	**FE Meta**	452	0.79	0.62-1.01	0.06	75.2	0.04	1.40	1.08-1.80	9.70×10^{-3}	84.2	0.01
	**RE Meta**		0.71	0.41-1.24	0.23			1.65	0.78-3.48	0.19		
**SCZA**	FOR2107	48	0.52	0.23-1.19	0.12			3.06	1.09-8.55	0.03		
	PsyCourse	67	0.92	0.51-1.68	0.79			0.94	0.51-1.73	0.84		
	**FE Meta**	115	0.76	0.47-1.23	0.26	16.8	0.27	1.28	0.76-2.15	0.36	73.2	0.05
	**RE Meta**		0.75	0.43-1.28	0.29			1.57	0.50-4.94	0.44		
**SCZ**	FOR2107	92	0.63	0.39-1	0.05			2.25	1.36-3.73	1.64×10^{-3}		
	PsyCourse	273	1.05	0.80-1.38	0.72			1.17	0.89-1.52	0.27		
	**FE Meta**	365	0.92	0.73-1.17	0.51	71.4	0.06	1.35	1.06-1.71	0.01	80.4	0.02
	**RE Meta**		0.84	0.51-1.39	0.5			1.56	0.82-2.96	0.17		
**ALL SAMPLES**	**FE Meta**	1786	0.78	0.70-0.87	$\mathbf{1.01\times10^{-5}}$	48.3	0.06	1.37	1.23-1.54	$\mathbf{2.11\times10^{-8}}$	57.9	0.02
	**RE Meta**		0.76	0.63-0.91	3.77×10^{-3}			1.49	1.19-1.85	$\mathbf{3.82\times10^{-4}}$		

Abbreviations: BD, bipolar disorder; FE Meta, inverse variance-weighted fixed-effects meta-analysis; MDD, major depressive disorder; 95% CI, 95% confidence intervals (the 95% CIs were constrained to a minimum of 0 and a maximum of 1); OR, odds ratio (a higher OR indicates an association with suicidal Ideation; RE Meta, inverse variance-weighted random-effects meta-analysis; SCZ, schizophrenia; SCZA, schizoaffective disorder; *p*, unadjusted *p* value (significance threshold corrected for multiple testing by Bonferroni's method: $\alpha = 3.57\times10^{-3}$; significant adjusted *p* values are indicated in bold font).

Funding, conflict of interest and acknowledgements

Romania1 and Romania2:
Recruitment and genotyping of the Romanian samples was supported by multiple grants from UEFISCDI including grant PN-III-P4-ID-PCE-2020-2269 (203/2021)

PsyCourse:
SCHU 1603/4-1, 5-1, 7-1, FA241/16-1

FOR2107:
This work is part of the German multi-center consortium "Neurobiology of Affective Disorders. A translational perspective on brain structure and function", funded by the German Research Foundation (Deutsche Forschungsgemeinschaft DFG; Forschungsgruppe/Research Unit FOR2107). The Forschungsgruppe/Research Unit FOR2107 study was funded by the German Research Foundation (DFG): grants KI 588/14-1, KI 588/14-2 to TK; DA 1151/5-1, DA 1151/5-2 to UD; NE 2254/12 to IN; HA 7070/2-2, HA 7070/3, HA 7070/4 to TH; RI 908/11-1, RI 908/11-2 to MR; NO 246/10-1, NO 246/10-2 to MMN and WI3439/3-2 to SHW. The study was supported by the German Federal Ministry of Education and Research (BMBF), through the Integrated Network IntegraMent, under the auspices of the e:Med programme (grants 01ZX1314A, 01ZX1614A to MMN; 01ZX1314G, 01ZX1614G to MR), through BMBF grants 01EE1406C to and 01EE1409C to MR, and through ERA-NET NEURON, "SynSchiz - Linking synaptic dysfunction to disease mechanisms in schizophrenia - a multilevel investigation" (01EW1810 to MR), and "EMBED" (grant 01EW1904).
This work is part of the German multicenter consortium "Neurobiology of Affective Disorders. A translational perspective on brain structure and function," funded by the German Research Foundation (Deutsche Forschungsgemeinschaft DFG; Forschungsgruppe/Research Unit FOR2107). Principal investigators (PIs) and their respective areas of responsibility in the FOR2107 consortium are as follows:
Work Package (WP) 1, FOR2107/MACS cohort and brain imaging: Tilo Kircher (speaker FOR2107; DFG grant numbers KI 588/14-1, KI 588/14-2), Udo Dannlowski (co-speaker FOR2107; DA 1151/5-1, DA 1151/5- 2), Axel Krug (KR 3822/5-1, KR 3822/7-2), Igor Nenadic (NE 2254/1-2), and Carsten Konrad (KO 4291/3- 1).
WP2, animal phenotyping: Markus Wöhr (WO 1732/4-1, WO 1732/4-2) and Rainer Schwarting (SCHW 559/14-1, SCHW 559/14-2).
WP3, miRNA: Gerhard Schratt (SCHR 1136/3-1, 1136/3-2).
WP4, immunology, mitochondria: Judith Alferink (AL 1145/5-2), Carsten Culmsee (CU 43/9-1, CU 43/9-2), and Holger Garn (GA 545/5-1, GA 545/7-2).
WP5, genetics: Marcella Rietschel (RI 908/11-1, RI 908/11-2), Markus Nöthen (NO 246/10-1, NO 246/10-2), and Stephanie Witt (WI 3439/3-1, WI 3439/3-2).
WP6, multimethod data analytics: Andreas Jansen (JA 1890/7-1, JA 1890/7-2), Tim Hahn (HA 7070/2-2), Bertram Müller-Myhsok (MU1315/8-2), Astrid Dempfle (DE 1614/3-1, DE 1614/3-2). CP1, biobank: Petra Pfefferle (PF 784/1-1, PF 784/1-2), Harald Renz (RE 737/20-1, 737/20-2). CP2, administration. Tilo Kircher (KI 588/15-1, KI 588/17-1), Udo Dannlowski (DA 1151/6-1), and Carsten Konrad (KO 4291/4-1).
Data access and responsibility: All PIs take responsibility for the integrity of the respective study data and their components. All authors and coauthors had full access to all study data.
Acknowledgements and members by Work Package (WP):
WP1: Henrike Bröhl, Katharina Brosch, Bruno Dietsche, Rozbeh Elahi, Jennifer Engelen, Sabine Fischer, Jessica Heinen, Svenja Klingel, Felicitas Meier, Tina Meller, Julia-Katharina Pfarr, Kai Ringwald, Torsten Sauder, Simon Schmitt, Frederike Stein, Annette Tittmar, and Dilara Yüksel (Dept. of Psychiatry, Marburg University); Mechthild Wallnig and Rita Werner (Core-Facility Brainimaging, Marburg University); Carmen Schade-Brittinger and Maik Hahmann (Coordinating Centre for Clinical Trials, Marburg); Michael Putzke (Psychiatric Hospital, Friedberg); Rolf Speier and Lutz Lenhard (Psychiatric Hospital, Haina); Birgit Köhnlein (Psychiatric Practice, Marburg); Peter Wulf, Jürgen

Kleebach, and Achim Becker (Psychiatric Hospital Hephata, Schwalmstadt- Treysa); Ruth Bär (Care Facility Bischoff, Neukirchen); Matthias Müller, Michael Franz, Siegfried Scharmann, Anja Haag, Kristina Spenner, and Ulrich Ohlenschläger (Psychiatric Hospital Vitos, Marburg); Matthias Müller, Michael Franz, and Bernd Kundermann (Psychiatric Hospital Vitos, Gießen); Christian Bürger, Katharina Dohm, Fanni Dzvonyar, Verena Enneking, Stella Fingas, Katharina Förster, Janik Goltermann, Dominik Grotegerd, Hannah Lemke, Susanne Meinert, Nils Opel, Ronny Redlich, Jonathan Repple, Kordula Vorspohl, Bettina Walden, and Dario Zaremba (Dept. of Psychiatry, University of Münster); Harald Kugel, Jochen Bauer, Walter Heindel, and Birgit Vahrenkamp (Dept. of Clinical Radiology, University of Münster); Gereon Heuft and Gudrun Schneider (Dept. of Psychosomatics and Psychotherapy, University of Münster); Thomas Reker (LWL-Hospital Münster); Gisela Bartling (IPP Münster); and Ulrike Buhlmann (Dept. of Clinical Psychology, University of Münster).
WP5: Helene Dukal, Christine Hohmeyer, Lennard Stütz, Viola Lahr, Fabian Streit, Josef Frank, and Lea Sirignano (Dept. of Genetic Epidemiology, Central Institute of Mental Health, Medical Faculty Mannheim, Heidelberg University); Stefanie Heilmann-Heimbach, Stefan Herms, and Per Hoffmann (Institute of Human Genetics, University of Bonn, School of Medicine & University Hospital Bonn); and Andreas J. Forstner (Institute of Human Genetics, University of Bonn, School of Medicine & University Hospital Bonn; Centre for Human Genetics, Marburg University).
WP6: Anastasia Benedyk, Miriam Bopp, Roman Keßler, Maximilian Lückel, Verena Schuster, and Christoph Vogelbacher (Dept. of Psychiatry, Marburg University); Jens Sommer and Olaf Steinsträter (Core-Facility Brain Imaging, Marburg University); and Thomas WD Möbius (Institute of Medical Informatics and Statistics, Kiel University).
CP1: Julian Glandorf, Fabian Kormann, Arif Alkan, Fatana Wedi, Lea Henning, Alena Renker, Karina Schneider, Elisabeth Folwarczny, Dana Stenzel, Kai Wenk, Felix Picard, Alexandra Fischer, Sandra Blumenau, Beate Kleb, Doris Finholdt, Elisabeth Kinder, Tamara Wüst, Elvira Przypadlo, and Corinna Brehm (Comprehensive Biomaterial Bank Marburg, Marburg University).

The FOR2107 cohort project (WP1) was approved by the Ethics Committees of the Medical Faculties, University of Marburg (AZ: 07/14) and University of Münster (AZ: 2014-422-b-S). The study was supported by the German Federal Ministry of Education and Research (BMBF) through the Integrated Network IntegraMent under the auspices of the e:Med programme (grants 01ZX1314A/01ZX1614A to MMN; 01ZX1314G/01ZX1614G to MR; 01ZX1614J to BMM), through grants 01EE1406C to MR and 01EE1409C to MR and SHW, and through ERA-NET NEURON, "SynSchiz - Linking synaptic dysfunction to disease mechanisms in schizophrenia - a multilevel investigation" (01EW1810 to MR) and BMBF grants 01EE1409C and 01EE1406C to MR and SHW.

Poland:
The database keeping for archived samples was founded by Poznan University of Medical Sciences sources: 502-20-22196440.

Igor Nenadic: NE 2254/1-2, NE 2254/3-1, NE 2254/4-1

Tadafumi Kato: Dr. Kato reports grants and personal fees from Japan Agency for Medical Research and Development (AMED), grants and personal fees from Ministry of Education, Culture, Sports, Science and Technology (MEXT)/Japan Society for the Promotion of Science (JSPS), personal fees from Kyowa Hakko Kirin Co., Ltd., personal fees from Eli Lilly Japan K.K., grants and personal fees from Otsuka Pharmaceutical Co., Ltd., personal fees from GlaxoSmithKline K.K., personal fees from Taisho Pharma Co., Ltd., grants and personal fees from Dainippon Sumitomo Pharma Co., Ltd., personal fees from Meiji Seika Pharma Co., Ltd., personal fees from Pfizer Japan Inc., personal fees from Mochida Pharmaceutical Co., Ltd., grants and personal fees from Shionogi & Co., Ltd., personal fees from Janssen Pharmaceutical K.K., personal fees from Janssen Asia Pacific, personal fees from Yoshitomiyakuhin, personal fees from Astellas Pharma Inc., personal fees from Nippon Boehringer Ingelheim Co. Ltd., personal fees from MSD K.K., personal fees from Kyowa Pharmaceutical Industry Co., Ltd., grants and personal fees from Takeda Pharmaceutical Co., Ltd., personal fees from Taisho Pharmaceutical Co., Ltd., personal fees from Taisho Toyama Pharmaceutical Co., Ltd., grants and

personal fees from Eisai Co., Ltd., grants and personal fees from Mitsubishi Tanabe Pharma Corporation, grants from Teijin Pharma, outside the submitted work.

References for the Online Resource

1. Mullins N, Forstner AJ, O'Connell KS, et al (2021) Genome-wide association study of more than 40,000 bipolar disorder cases provides new insights into the underlying biology. Nat Genet. https://doi.org/10.1038/s41588-021-00857-4
2. Kircher T, Wöhr M, Nenadic I, et al (2019) Neurobiology of the major psychoses: a translational perspective on brain structure and function—the FOR2107 consortium. Eur Arch Psychiatry Clin Neurosci 269:949–962. https://doi.org/10.1007/s00406-018-0943-x
3. Budde M, Anderson-Schmidt H, Gade K, et al (2019) A longitudinal approach to biological psychiatric research: The PsyCourse study. Am J Med Genet B Neuropsychiatr Genet 180:89–102. https://doi.org/10.1002/ajmg.b.32639
4. Lam M, Awasthi S, Watson HJ, et al (2020) RICOPILI: Rapid Imputation for COnsortias PIpeLIne. Bioinformatics 36:930–933. https://doi.org/10.1093/bioinformatics/btz633
5. Delaneau O, Zagury J-F, Marchini J (2013) Improved whole-chromosome phasing for disease and population genetic studies. Nat Methods 10:5–6. https://doi.org/10.1038/nmeth.2307
6. Howie BN, Donnelly P, Marchini J (2009) A Flexible and Accurate Genotype Imputation Method for the Next Generation of Genome-Wide Association Studies. PLoS Genet 5:e1000529. https://doi.org/10.1371/journal.pgen.1000529
7. Howie B, Fuchsberger C, Stephens M, et al (2012) Fast and accurate genotype imputation in genome-wide association studies through pre-phasing. Nat Genet 44:955–959. https://doi.org/10.1038/ng.2354
8. Chang CC, Chow CC, Tellier LC, et al (2015) Second-generation PLINK: rising to the challenge of larger and richer datasets. Gigascience 4:7. https://doi.org/10.1186/s13742-015-0047-8
9. McCarthy S, Das S, Kretzschmar W, et al (2016) A reference panel of 64,976 haplotypes for genotype imputation. Nat Genet 48:1279–1283. https://doi.org/10.1038/ng.3643
10. Ge T, Chen CY, Ni Y, et al (2019) Polygenic prediction via Bayesian regression and continuous shrinkage priors. Nat Commun 10:1–10. https://doi.org/10.1038/s41467-019-09718-5
11. Berg SM van den, Moor MHM de, Verweij KJH, et al (2016) Meta-analysis of Genome-Wide Association Studies for Extraversion: Findings from the Genetics of Personality Consortium. Behav Genet 46:170. https://doi.org/10.1007/S10519-015-9735-5
12. Luciano M, Hagenaars SP, Davies G, et al (2018) Association analysis in over 329,000 individuals identifies 116 independent variants influencing neuroticism. Nat Genet 50:6–11. https://doi.org/10.1038/s41588-017-0013-8
